# Whole-genome sequencing for the characterization of resistance mechanisms and epidemiology of colistin-resistant *Acinetobacter baumannii*

**DOI:** 10.1371/journal.pone.0264335

**Published:** 2022-03-04

**Authors:** Chorong Hahm, Hae-Sun Chung, Miae Lee

**Affiliations:** 1 Department of Laboratory Medicine, College of Medicine, Ewha Womans University, Seoul, Korea; 2 Department of Laboratory Medicine, Eone Laboratories, Incheon, Korea; 3 EWHA Education and Research Center for Infection, College of Medicine, Ewha Womans University, Seoul, Korea; Nitte University, INDIA

## Abstract

**Background:**

Multidrug-resistant *Acinetobacter baumannii* is an important causal pathogen of healthcare-associated infections, and colistin-resistant strains have recently emerged owing to the increased use of colistin. Using next-generation sequencing (NGS), a single whole-genome sequencing (WGS) protocol can identify and type pathogens, analyze genetic relationships among different pathogens, predict pathogenic transmissions, and detect antibiotic resistance genes. However, only a few studies have applied NGS in studying the resistance mechanism and epidemiology of colistin-resistant *A*. *baumannii*. This study aimed to elucidate the resistance mechanism of colistin-resistant *A*. *baumannii* and analyze its molecular epidemiology through WGS.

**Materials and methods:**

The subjects in this study were patients who visited a university hospital between 2014 and 2018. Thirty colistin-resistant strains with high minimum inhibitory concentrations were selected from various patient samples, and WGS was performed. Comparative genomic analysis was performed for the 27 colistin-resistant *A*. *baumannii* strains using a colistin-susceptible strain as the reference genome.

**Results:**

The WGS analysis found no mutation for *lpxA*, *lpxC*, *lpx D*, *pmrA*, *pmrB*, and *mcr1*, the genes known to be associated with colistin resistance. Fifty-seven coding sequences (CDS) showed differences; they included 13 CDS with known names and functions that contained 21 genes. From the whole-genome multi-locus sequence typing (wgMLST) and single nucleotide polymorphism (SNP) analyses, two major clusters were found for the colistin-resistant *A*. *baumannii* strains. However, no differences were observed by the time of detection for each cluster, the samples, the pattern of antibiotic resistance, or the patient characteristics. In the conventional MLST following the Oxford scheme, the typing result showed ST1809, ST451, ST191, ST1837, and ST369 in the global clone 2 (GC2), without any relation with the results of wgMLST and SNP analyses.

**Conclusion:**

Based on the findings of the resistance gene analysis through WGS and comparative genomic analysis, the potential genes associated with colistin-resistance or CDS were examined. Furthermore, the analysis of molecular epidemiology through WGS regarding colistin-resistant *A*. *baumannii* may prove helpful in preventing infection by multidrug-resistant bacteria and controlling healthcare-associated infections.

## Introduction

*Acinetobacter baumannii* is a widely distributed species in the hospital environment, and as an important causal pathogen of healthcare-associated infections, the species causes infection outbreaks in hospitals. If a patient with a severe underlying disease is affected, the reported mortality rate is substantially higher than that of other patients. In particular, the frequency of multidrug-resistant *A*. *baumannii* strains with resistance to three or more types of antibiotics has increased considerably lately [1].

The outbreak of *A*. *baumannii* is frequently reported worldwide; the reported incidence of multidrug-resistant (MDR) *A*. *baumannii* has increased in regions including Europe, North America and Asia. Treatment with broad-spectrum antibiotics often leads to MDR *A*. *baumannii* infection in patients with severe underlying diseases or reduced immunity and those who have undergone invasive surgery. This could lead to ventilator-related pneumonia, urinary tract infection, or sepsis, especially in the intensive care unit (ICU), and cause treatment failure, consequent complications, and increased mortality by preventing the selection of a suitable antibiotic for the treatment.

Colistin shows a relatively high rate of cure against serious infections by multidrug-resistant gram-negative bacteria resulting in a steady increase in its use. As a positively charged peptide, colistin acts by binding to the extracellular lipopolysaccharide (LPS) in gram-negative bacilli, displacing Mg^2+^ and Ca^2+^ crosslinked to it and destabilizing LPS molecules on the cell membrane. This increases the bacterial cell membrane permeability, leading to increased colistin uptake, resulting in the leakage of intracellular contents of the cell and cell death. Thus, a mutation of LPS can lead to resistance by preventing the action of colistin on the cell membrane [2]. Recently, a multidrug-resistant *A*. *baumannii* strain with colistin resistance has been reported [3]. The percentage of colistin-resistant *A*. *baumannii* is relatively low (1–3%) worldwide [4]; nonetheless, as colistin resistance indicates pan-drug resistance that shows resistance to all antibiotics in most cases, the lack of a suitable antibiotic for treatment has become a serious problem. It is thus crucial in the control of severe healthcare-associated infections that the resistance mechanism of the multidrug-resistant *A*. *baumannii* to colistin be elucidated to prevent the spread of the multidrug-resistant *A*. *baumannii*.

Next-generation sequencing (NGS) is a method of analysis where the genome is decoded by dividing it into myriads of fragments and combining each of their sequences. Compared to the conventional Sanger sequencing, NGS offers more rapid analysis, and as a single protocol, it can identify and type pathogens, analyze genetic relationships among different pathogens, predict pathogenic transmissions, and detect antibiotic resistance genes; this method may prove to be valuable for testing and studying clinical microbiology. However, only a few studies have applied NGS in studying the resistance mechanism and epidemiology of colistin-resistant *A*. *baumannii* [5].

The purpose of this study is to elucidate the resistance mechanism of colistin-resistant *A*. *baumannii* and analyze its molecular epidemiology through NGS-based whole-genome sequencing.

## Subjects and methods

### A. Subjects

The subjects in this study were patients admitted to Ewha Womans University Mokdong Hospital between 2014 and 2018, from whom various *A*. *baumannii* samples were isolated. The VITEK2 system (bioMerieux, Marcy l’Etoile, France) or VITEK MS (bioMerieux, Marcy l’Etoile, France) was used for species identification, and the strains that showed colistin resistance (minimum inhibitory concentration [MIC] ≥ 4 μg/mL) as the result of antibiotic susceptibility using the VITEK2 system, were collected. The duplicate strains were excluded, and the percentage of colistin resistance in *A*. *baumannii* between 2014 and 2018 was 1.9% (84/4467). Among them, whole-genome sequencing was performed for the 30 colistin-resistant *A*. *baumannii* strains that showed high MIC (≥ 8μg/mL) on the broth microdilution test, and the strains were thus named S1–S30. The present study was conducted following the approval of the Institutional Review Board of Ewha Womans University Mokdong Hospital (EUMC 2019-09-013-003). As this study used residual samples, the risk to the subjects was extremely low, and it was a retrospective study, so the Institutional Review Board exempted the participants from filling out the consent form. In addition, all patient medical records and specimen information were anonymized, and individual identification or potential identification information of each patient was not included in this study.

### B. Methods

#### 1. Colistin resistance test

The antibiotic susceptibility test was performed using the N225 CARD (bioMerieux, Marcy l’Etoile, France) of the VITEK2 system for the following antibiotics: amikacin, ampicillin/sulbactam, cefepime, cefotaxime, ceftazidime, ciprofloxacin, colistin, gentamicin, imipenem, meropenem, minocycline, piperacillin, ticarcillin/clavulanic acid, and trimethoprim/sulfamethoxazole. In addition, the MIC for colistin was determined using the GNX3F plate of the Sensititre system (Thermo Fisher Scientific, Waltham, MA, USA) via the commercial broth microdilution kit and the standard broth microdilution. The breakpoint of the Clinical Laboratory Standard Institute (CLSI) M100-ED29 guideline was used to determine colistin resistance and susceptibility; MIC ≥4 μg/mL was taken to indicate colistin resistance. Reference MICs for colistin were determined using manual broth microdilution (BMD), performed according to the CLSI M07-ED11 guideline. MIC panels were prepared with pure colistin sulfate powder (Sigma–Aldrich, St. Louis, MO, USA) with two-fold dilutions in the range of 0.125–64 μg/mL in cation-adjusted MH broth (Becton–Dickinson, Sparks, MD, USA) in polystyrene plates (Greiner, Frickenhausen, Germany). Results were read after incubation in an aerobic atmosphere at 35 ± 2°C for 20–24 h. The results were interpreted based on the MICs according to the M100-ED29 guidelines.

#### 2. Whole-genome sequencing

*A*. *Microbial species identification and resistance gene analysis*. The DNA library of the selected strains was prepared using the Ion Xpress plus fragment library kit (Thermo Fisher Scientific, Waltham, MA, USA). Whole-genome sequencing was performed using the Ion S5xl sequencer system(Thermo Fisher Scientific, Waltham, MA, USA) according to the manufacturer’s guidelines. The sequencing reads were assembled using the Ion torrent suit software 5.10(Thermo Fisher Scientific, Waltham, MA, USA). To prevent cross-contamination, the ContEst16S tool v1.0 was used to compare the strains with other copies of 16s rRNA in the genome, and for species identification based on the genome, the TrueBac ID system (v1.92, DB:20190603) [https://www.truebacid.com/] was used [6, 7]. Each genome was combined with the genome of *A*. *baumannii*-type strain, and the Average Nucleotide Identify (ANI) was confirmed to be above the threshold value (≥ 95%). Prodigal v2.6.3 was used for gene-finding, and for gene-annotation, homology search (USEARCH v8.1.1861) was performed with the EggNOG v4.1, SEED 2015-12-10, Swiss-Prot [version 2015-12-10] and the KEGG databases [version 2018-10-01], while the variable was -accel 1.0 -evalue 1.0E-5 -maxaccepts 1. For the antibiotic resistance gene search, the Resistance Gene Identifier (RGI) v4.2.0 tool of the CARD database v3.0.2 was used [8–11].

*B*. *Comparative genomic analysis*. For comparative genomic analysis, the genome sequences with close association with *A*. *baumannii* were identified using the NCBI genome database. To compare the colistin-susceptible strains and the colistin-resistant strains, the genome of the colistin-susceptible *A*. *baumannii* ATCC 17978 was downloaded from the NCBI genome database. To understand the phylogenetic relationship among the strains to be analyzed, the OrthoANI was calculated and using the Orthologous ANI Tool (ChunLab, Seoul, Korea), the UPGMA dendrogram was produced. For the genome synteny analysis based on the nucleotide unit, the Blast Ring Image Generator (BRIG) was used with default variables [12, 13]. To analyze the pan-genome and core-genome, the Comparative Genomics (CG) pipeline of ezbiocloud Apps (https://www.ezbiocloud.net/apps, ChunLab Inc.) was used. To define the pan-genome orthologous groups (POGs), two methods were combined; i) reciprocal best hit using the uBLAST with *E*-value cut-off at 1×10^−6^ [14]; ii) independent open reading frame (ORF) method with at least 70% gene coverage as the threshold of the nucleotide sequence [15]. The magnitude of the pan-genome and core-genome was expressed as the number of orthologous genes on the Venn diagram and using the ezbiocloud CG pipeline, the heat-map was produced to indicate the presence of a given gene. For 2D metabolite biosynthetic gene cluster and antibiotic resistance gene analyses, the antiSMASH [16] and Antibiotic Resistance Genes Database (ARDB) web servers [17] were used. If a given gene was presumed to be a virulence gene, a keyword search using the annotated gene or an amino acid sequence homology search for the predicted virulence protein of *A*. *baumannii* was performed.

*C*. *Whole-genome multilocus sequence typing*. Roary v3.12.0 pipeline was used to produce the core gene set from the 23 complete *A*. *baumannii* genomes [18]. The aforementioned core gene was used to produce the species-specific reference genome named *A*. *baumannii*. To conduct wgMLST, the core gene set discovered during the production of the species-specific standard sequence was used. For each core gene, the hidden Markov model (HMMs) was produced using the MAFFT v7.3.10 [19] and nhmmer v3.1b2 tool. The core gene was identified using the HMM-based search and was used to determine the number of unique alleles in each gene. For the minimum spanning tree based on wgMLST, coding was performed with the Kruskal algorithm for the in-house JavaScript (ChunLab, Seoul, Korea) [20].

In addition, the data of whole-genome sequencing were used, and based on the data of seven housekeeping genes (*gltA*, *gyrB*, *gdhB*, *recA*, *cpn60*, *gpi*, and *rpoD*), the sequence types were identified via conventional MLST using the Oxford scheme [21].

*D*. *Single nucleotide polymorphism analysis*. To calculate the genome sequence SNVs of the final dataset, the MUMmer v3.23 program was used, while applying the ATCC 19606 strain as the standard sequence [22]. The multiple sequence alignment was edited based on the detected SNPs, and the maximum likelihood phylogenetic tree was drawn using the RAxML v8.2.11 with 1,000 bootstrap re-samplings and GTRCAT as the nucleotide model [23].

### C. Statistical analysis

To analyze the clinical characteristics of the clusters 1 and 2 from whole-genome multi-locus sequence typing (wgMLST), Student’s *t*-test or Mann-Whitney *U*-test was performed for age and the admission duration prior to *A*. *baumannii* isolation; Chi-square test or Fisher’s exact test was performed for gender, department of admission, underlying disease (diabetes, cancer, chronic renal disease, chronic liver disease, cardiovascular disease, gastrointestinal disease, and respiratory disease), history of admission within one year, history of antibiotic treatment within 28 days of *A*. *baumannii* isolation and the type of antibiotics used, ICU admission within 28 days, use of a ventilator within three months, central vein cannulation, tracheotomy, percutaneous abscess drainage, hemodialysis and other invasive procedures, co-infection with the isolation of a different strain from the same or different sample within three days of *A*. *baumannii* identification, and resistance to antibiotics. The significance was set at *P*<0.05. SPSS 23.0 software (IBM Corp, Armonk, NY, USA) was used for all statistical analyses.

## Results

### Whole-genome sequencing

#### A. Microbial species identification and resistance gene analysis

The result of analyzing 30 *A*. *baumannii* strains using the TrueBac ID system, following the whole-genome sequencing, showed that 27 strains were *A*. *baumannii*. In comparison, three strains (S1, S11, and S12) were *Acinetobacter colistiniresistens* and thus excluded from subsequent analyses of resistance mechanism and epidemiology. The resistance gene search was performed using the CARD database, and no mutation was detected for the genes *lpxA*, *lpxC*, *lpxD*, *pmrA*, *pmrB*, and *mcr1* that are known to be related to colistin resistance. The resistance gene search result for other antibiotics is presented in Table 1 and S1 Table. Regarding the genes associated with resistance to carbapenem-based agents, *adeI*, *adeJ*, *adeK*, *adeN*, *OXA-133*, *OXA-23*, *OXA-66*, and *TEM-1* were detected.

**Table 1:** Beta lactam resistance genes identified using whole-genome sequencing in 27 colistin-resistant *A*. *baumannii*.

AMR Gene Family	Gene name	Mechanism	Drug class	Number of isolates
ADC beta-lactamase	*ADC-18*	inactivation	cephalosporin	27
OXA beta-lactamase	*OXA-133*	inactivation	cephalosporin; penam	3
OXA beta-lactamase	*OXA-23*	inactivation	cephalosporin; penam	23
OXA beta-lactamase	*OXA-420*	inactivation	cephalosporin; penam	0
OXA beta-lactamase	*OXA-66*	inactivation	cephalosporin; penam	27
TEM beta-lactamase	*TEM-1*	inactivation	monobactam; cephalosporin; penam; penem	15
VIM beta-lactamase	*VIM-2*	inactivation	carbapenem; cephalosporin; cephamycin; penam; penem	0
resistance-nodulation-cell division (RND) antibiotic efflux pump	*adeI*	efflux	macrolide; fluoroquinolone; lincosamide; carbapenem; cephalosporin; tetracycline; rifamycin; diaminopyrimidine; phenicol; penem	27
resistance-nodulation-cell division (RND) antibiotic efflux pump	*adeJ*	efflux	macrolide; fluoroquinolone; lincosamide; carbapenem; cephalosporin; tetracycline; rifamycin; diaminopyrimidine; phenicol; penem	27
resistance-nodulation-cell division (RND) antibiotic efflux pump	*adeK*	efflux	macrolide; fluoroquinolone; lincosamide; carbapenem; cephalosporin; tetracycline; rifamycin; diaminopyrimidine; phenicol; penem	27
resistance-nodulation-cell division (RND) antibiotic efflux pump	*adeN*	efflux	macrolide; fluoroquinolone; lincosamid; carbapenem; cephalosporin; tetracycline; rifamycin; diaminopyrimidine; phenicol; penem	27

#### B. Comparative genomic analysis

For the 27 colistin-resistant *A*. *baumannii* strains, following the whole genome sequencing using the colistin-susceptible *A*. *baumannii* ATCC 17978 as the reference genome, a comparative genomic analysis was performed. Using the reference genome *A*. *baumannii* ATCC 17978 as the standard strain, a genetic difference was presumed if the given strain showed no-hit for the gene/ortholog. By comparing with the reference genome, the CDS with potential association with colistin resistance was examined. Fifty-seven CDS differed from the colistin-susceptible *A*. *baumannii* ATCC 17978 strain, among which 13 CDS had a known name and function and contained 21 genes (*cysH*, *udg*, *ureC*, *DNMT1*, *dcm*, *tmk*, *DTYMK*, *glxK*, *garK*, *CHO1*, *pssA*, *mdh*, *udg*, *mutY*, *TST*, *MPST*, *sseA*, *cobS*, *cobV*, *ENOm*, and *eno*) as shown in Table 2. The WGS analysis found no mutation for *lpxA*, *lpxC*, *lpx D*, *pmrA*, *pmrB*, and *mcr1*, the genes known to be associated with colistin resistance.

**Table 2:** The difference in CDS between *A*. *baumannii* ATCC 17978 and colistin-resistant *A*. *baumannii* using comparative genomic analysis.

CDS name	Gene	Product	Function
GCA_001593425.2_00314	*tmk*, *DTYMK*	dTMP kinase	ATP-binding; Kinase; Nucleotide biosynthesis; Nucleotide-binding; Transferase
GCA_001593425.2_00428	*glxK*, *garK*	Glycerate 2-kinase	ATP-binding; Kinase; Nucleotide-binding; Transferase
GCA_001593425.2_00529	*pgpB*	Phosphatidylglycero-phosphatase	
GCA_001593425.2_00569	*cysH*	Phosphoadenylyl-sulfate reductase (thioredoxin)	Cytoplasm; Oxidoreductase
GCA_001593425.2_00814	*CHO1*, *pssA*	CDP-diacylglycerol-serine O-phosphatidyltransferase	Cell membrane; Lipid biosynthesis; Lipid metabolism; Membrane; Phospholipid biosynthesis; Phospholipid metabolism; Transferase
GCA_001593425.2_00819	*mdh*	Malate dehydrogenase	NAD; Oxidoreductase; Tricarboxylic acid cycle
GCA_001593425.2_01179	*udg*	Uracil-DNA glycosylase	
GCA_001593425.2_01504	*mutY*	Adenine glycosylase	4Fe-4S; DNA damage; DNA repair; Glycosidase; Hydrolase; Iron; Iron-sulfur; Metal-binding
GCA_001593425.2_01557	*TST*, *MPST*, *sseA*	Thiosulfate sulfurtransferase	Cytoplasm; Repeat; Transferase
GCA_001593425.2_02434	*ureC*	Urease	Cytoplasm; Hydrolase; Metal-binding; Nickel
GCA_001593425.2_03177	*cobS*, *cobV*	Adenosylcobinamide-GDP ribazoletransferase	Cell inner membrane; Cell membrane; Cobalamin biosynthesis; Magnesium; Membrane; Transferase; Transmembrane; Transmembrane helix
GCA_001593425.2_03435	*ENO*, *eno*	Phosphopyruvate hydratase	Cytoplasm; Glycolysis; Lyase; Magnesium; Metal-binding; Secreted
GCA_001593425.2_03614	*DNMT1*,*dcm*	DNA (cytosine-5-)-methyltransferase	

#### C. wgMLST and SNP analyses

**Fig 1:**
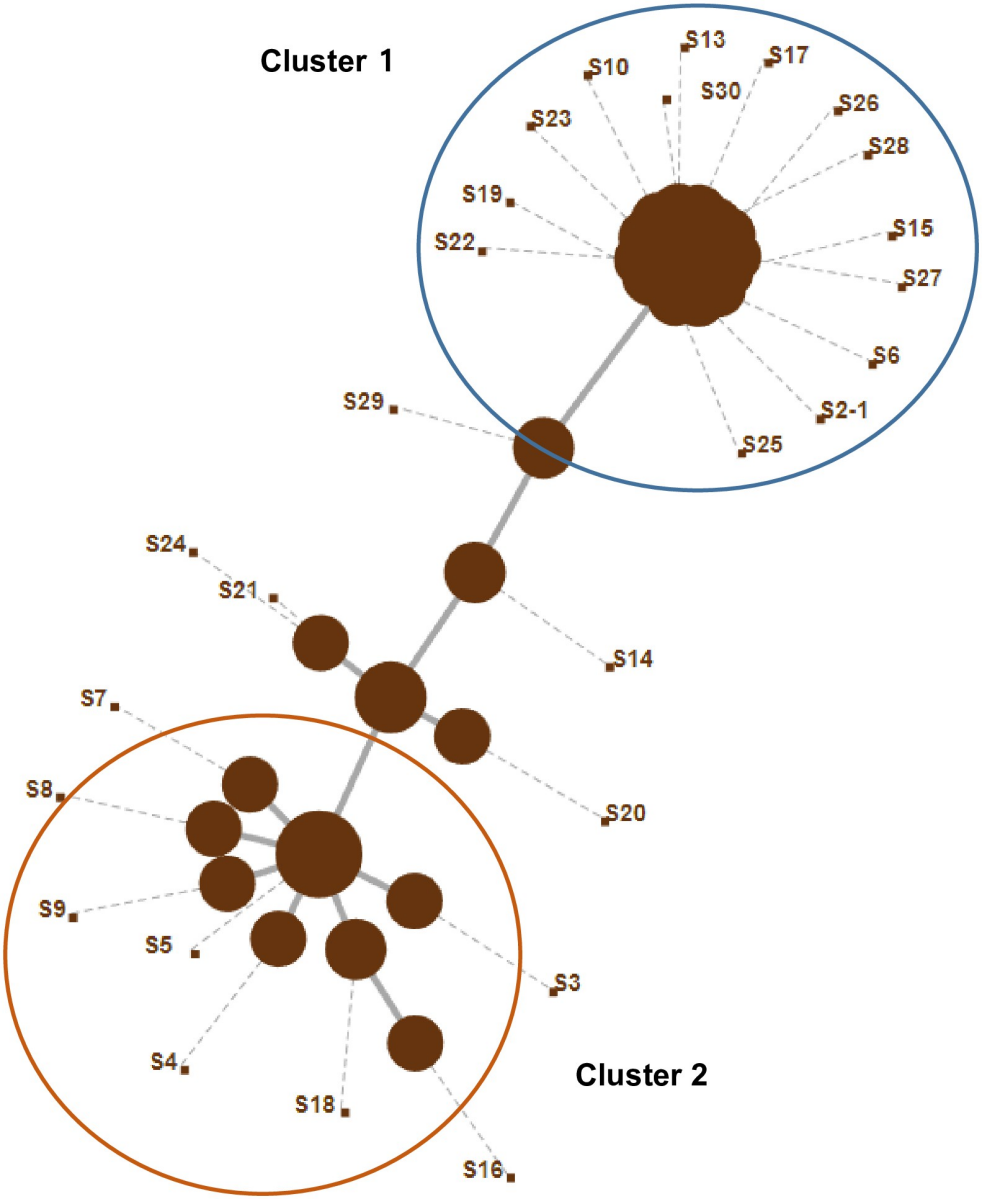
Results of 27 *A*. *baumannii* according to whole-genome multilocus sequence typing (wgMLST). Results of the wgMLST of colistin-resistant *A*. *baumannii* strains showed two major clusters.

**Fig 2:**
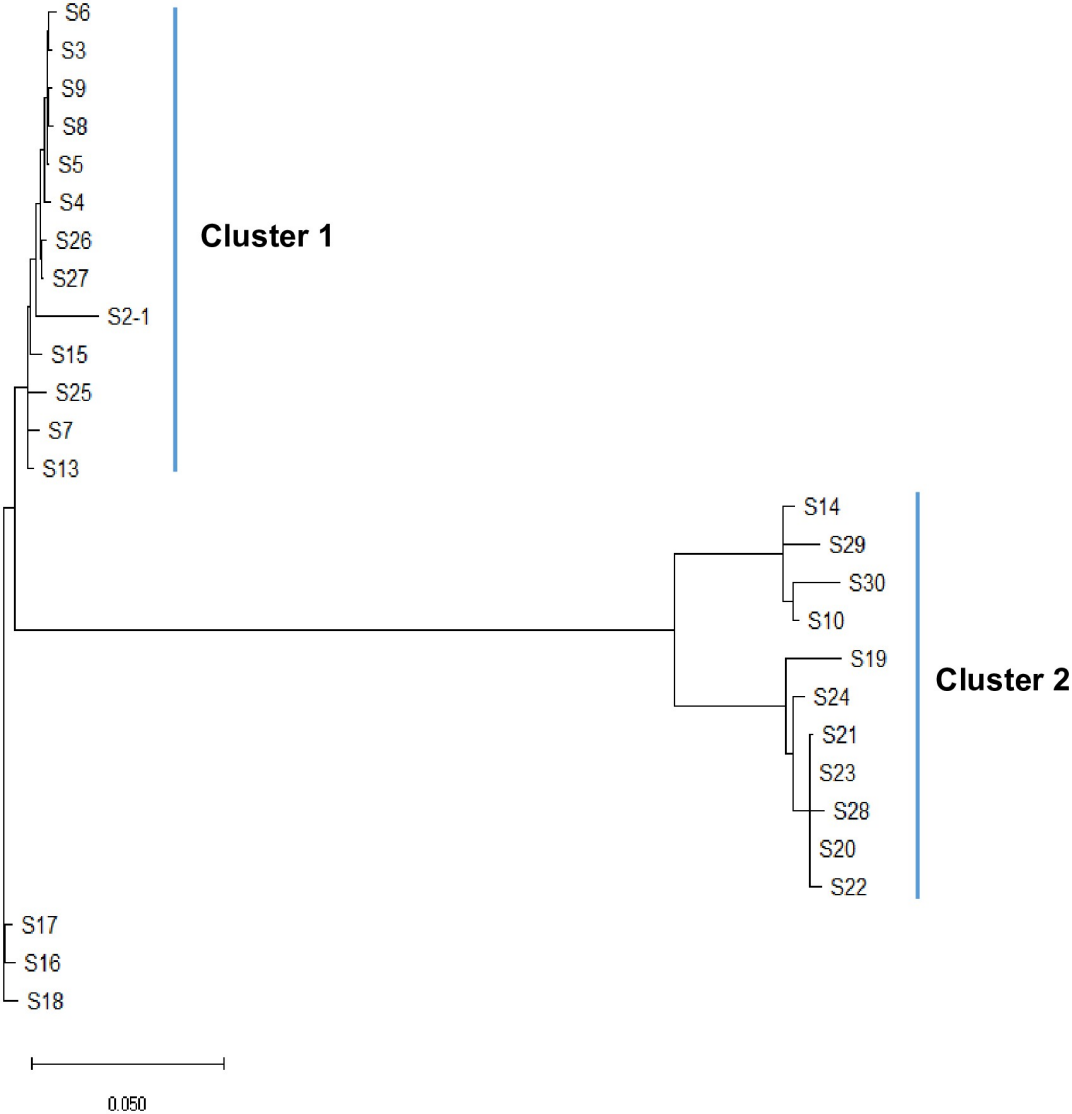
Phylogenetic trees of 27 *A*. *baumannii* according to single nucleotide polymorphism (SNP) analysis. Results of the phylogenetic tree using SNP analysis showed two major clusters.

The phylogenetic trees obtained through wgMLST and single nucleotide polymorphism (SNP) analyses are presented in Figs 1 and 2. The result of the wgMLST analysis showed two major clusters for the colistin-resistant *A*. *baumannii* strain (Fig 1); likewise, the result of SNP analysis showed two major clusters (Fig 2). Neither wgMLST nor SNP analyses found a difference in the time of detection for each cluster, the hospital ward, the samples, and the antibiotic resistance pattern (Tables 3 and 4). The conventional MLST using the data of seven housekeeping genes (*gltA*, *gyrB*, *gdhB*, *recA*, *cpn60*, *gpi*, and *rpoD*) led to typing ST1809, ST451, ST191, ST1837, and ST369, with no relation to wgMLST or SNP analyses (Table 5).

**Table 3:** Microbiologic characteristics of Cluster 1 and Cluster 2 according to whole-genome multilocus sequence typing (wgMLST).

Factor	Cluster1 (n = 14)	Cluster2 (n = 8)	p-Value
**Specimen**			0.425
Urine	2 (14.3%)		
Sputum	10 (71.4%)	8 (100%)	
CSF	1 (7.1%)		
Others	1 (7.1%)		
**Antimicrobial Resistance**			
Amikacin	12 (85.7%)	8 (100%)	0.907
Ampicillin/sulbactam	7 (50.0%)	5 (62.5%)	0.571
Cefepime	12 (85.7%)	8 (100%)	0.262
Cefotaxime	14 (100.0%)	8 (100%)	
Ceftazidime	14 (100.0%)	8 (100%)	
Ciprofloxacin	14 (100.0%)	8 (100%)	
Gentamicin	13 (92.9%)	7 (87.5%)	0.674
Imipenem	14 (100.0%)	8 (100%)	
Meropenem	14 (100.0%)	6 (75.0%)	0.050
Minocycline	5 (35.7%)	2 (25.0%)	0.457
Piperacillin	14 (100.0%)	8 (100%)	
Ticacillin/Clavulanic acid	14 (100.0%)	8 (100%)	
Trimethoprim/sulfamethoxazole	13 (92.9%)	7 (87.5%)	0.674

**Table 4:** Epidemiologic characteristics of Cluster 1 and cluster 2 according to whole-genome multilocus sequence typing (wgMLST).

Factor	Cluster1 (n = 14)	Cluster2 (n = 8)	p-Value
**Age, median (range)**	75 (1–89)	64 (48–88)	0.924
**Sex**			0.571
Male	7 (50.0%)	3 (37.5%)	
Female	7 (50.0%)	5 (62.5%)	
**Admission department**			0.389
Internal medicine	9 (64.3%)	5 (62.5%)	
General surgery	2 (14.3%)	1 (12.5%)	
Orthopedics	1 (7.1%)		
Neurology	2 (14.3%)		
Rehabilitation medicine		1 (12.5%)	
Other		1 (12.5%)	
**Co-morbidity**			
Diabetes mellitus	3 (21.4%)	1 (12.5%)	0.601
Cancer	3 (21.4%)	3 (37.5%)	0.262
Chronic kidney disease	4 (28.6%)	1 (12.5%)	0.387
Chronic liver disease	0 (0.0%)	2 (25.0%)	0.050
Cardiovascular disease	4 (28.6%)	2 (25.0%)	0.856
Gastro-intestinal disease	2 (14.3%)	1 (12.5%)	0.907
Pulmonary disease	2 (14.3%)	1 (12.5%)	0.907
**Length of hospital stay before *A*. *baumannii* infection (day)**	47	29	0.306
**Hospitalization**			
Last 1 year	7 (28.6%)	3 (37.5%)	0.665
Previous antibiotic use (last 28 days)	2 (14.3%)	0 (0.0%)	0.262
ICU stay	6 (42.9%)	5 (62.5%)	0.375
**Invasive procedure**			
Mechanical ventilation	9 (64.3%)	3 (37.5%)	0.225
Central venous catheter	6 (42.9%)	2 (25.0%)	0.402
Tracheostomy	3 (21.4%)	3 (37.5%)	0.416
Percutaneous drainage	6 (11.8%)	1 (12.5%)	0.907
Hemodialysis	4 (28.6%)	2 (25.0%)	0.856
**Previous antibiotic use**			
Cephalosporin	1 (7.1%)	2 (25.0%)	0.240
Fluoroquinolone	1 (7.1%)	1 (12.5%)	0.674
Carbapenem	5 (35.7%)	4 (50.0%)	0.512
Aminoglycoside	2 (14.3%)	0 (0.0%)	0.262
Penicillin	8 (57.1%)	0 (0.0%)	0.806
Colistin	1 (7.1%)	1 (12.5%)	0.674
Vancomycin	6 (42.9%)	2 (25.0%)	0.402
**Co-infection** *	12 (85.7%)	5 (62.5%)	0.211

* Other bacteria are isolated from the same or other samples within 3 days of *A*. *baumannii* isolation.

**Table 5:** Description of 22 patients and isolates associated with two clusters of colistin-resistant *A*. *baumannii*.

Cluster by wg-MLST	Cluster by SNP	ST type based on MLST obtained by sequencing result of 7 housekeeping gene	Isolates No.	Time of Isolation from admission (day)	Days for Hospital stay	Ward at isolation	ICU stay before isolation
1	1	ST451/ST1809	S2-1	14	2014.01.28–2014.03.06	1ICU	3ICU, 1ICU
	1	ST451/ST1809	S6	5	2014.10.23–2014.11.17	2ICU	2ICU
	2	ST191	S10	28	2015.03.25–2015.04.28	52ward	no
	1	ST191	S13	142	2015.07.05–2016.01.05	62ward	3ICU
	1	ST451/ST1809	S15	156	2016.02.09–2016.07.16	102ward	no
	-	ST451/ST1809	S17	15	2016.08.22–2016.10.04	62ward	no
	2	ST369/ST1837	S19	7	2017.09.22–2017.09.28	2ICU	2ICU
	2	ST369/ST1837	S22	15	2017.09.23–2017.11.11	2ICU	2ICU
	2	ST369/ST1837	S23	36	2017.09.04–2017.12.23	3ICU	3ICU
	1	ST451/ST1809	S25	23	2017.09.24–2017.12.16	62ward	no
	1	ST451/ST1809	S26	43	2018.02.16–2018.04.01	SICU	SICU
	1	ST451/ST1809	S27	23	2018.07.12–2018.08.03	62ward	no
	2	ST369/ST1837	S28	56	2018.07.10–2017.12.04	82ward	no
	2	ST191	S30	27	2018.09.26–2018.11.11	102ward	no
2	1	ST451/ST1809	S3	7	2014.08.13–2014.09.25	62ward	1ICU, 2ICU
	1	ST451/ST1809	S4	19	2014.08.24–2015.01.05	2ICU	2ICU
	1	ST451/ST1809	S5	53	2014.09.09–2014.11.10	2ICU	2ICU
	1	ST451/ST1809	S7	15	2014.12.03–2014.12.31	71ward	2ICU
	1	ST451/ST1809	S8	12	2014.11.20–2015.01.05	1ICU	1ICU
	1	ST451/ST1809	S9	54	2014.11.21–2015.01.17	61ward	1ICU
	-	ST451/ST1809	S16	59	2016.06.11–2016.07.15	82ward	no
	-	ST451/ST1809	S18	65	2016.10.09–2016.12.09	71ward	no
-	2	ST191	S14	13	2016.05.18–2016.05.30	53ward	no
-	2	ST369/ST1837	S20	33	2017.08.27–2017.1120	1ICU	1ICU
-	2	ST369/ST1837	S21	65	2017.07.26–2017.12.03	1ICU	1ICU
-	2	ST369/ST1837	S24	14	2017.09.26–2017.10.17	2ICU	2ICU
-	2	ST191	S29	12	2018.09.04–2018.09.26	3ICU	3ICU, SICU

## Discussion

The most critical mechanism in the acquisition of carbapenem resistance by *Acinetobacter* is the production of carbapenemase that can hydrolyze carbapenem. Additionally, non-enzymatic mechanisms can contribute to carbapenem resistance by altering the outer membrane protein, modifying the affinity or expression of the penicillin-binding protein, or by overexpressing the efflux pump. The genes associated with carbapenem resistance identified in this study are *adeI*, *adeJ*, *adeK*, *adeN*, *OXA-133*, *OXA-23*, *OXA-66*, *and TEM-1*. These genes were involved in the mechanism of carbapenemase production and the overexpression of the efflux pump. When the resistance mechanism of carbapenem-resistant *A*. *baumannii* was investigated using PCR at a university hospital in Korea, and the result showed that the following genes were related to carbapenem resistance; *OXA-51* (100%), *OXA-23* (100%), *AmpC* (100%), *PER-1* (13.0%), *arm-A* (84.1%), *aacA4* (95.7%), *aacC1* (81.2%), *aphA6* (15.9%), and *aphA1* (8.7%) [24]. In this study, several other genes, including *OXA-23* (76.7%), *arm-A* (80.0%), and *aacA4* (95.7%), were found to be associated with carbapenem resistance in the Korean strains.

The colistin resistance mechanism of *A*. *baumannii* has not yet been elucidated. As in the colistin resistance mechanism of other gram-negative bacteria, it is likely that the resistance is acquired by activating the LPS modification operon through the mutations of *pmrA* and *pmrB*, the two-component regulatory system, while the mutations of the genes essential in lipid A biosynthesis *(lpxA*, *lpxC* or *lpxD*) may also relate [25, 26]. In a study based on whole-genome sequencing, 21 colistin-resistant *A*. *baumannii* strains were examined, and *pmrAB* mutation was detected in 71.4%, while LPS mutation was not observed [27]. In another study based on Sanger sequencing, 17 colistin-resistant *A*. *baumannii* strains were examined, and *lpxA* (29.4%), *lpxC* (29.4%), and *lpxD* (52.9%) were detected [28]. In a recent study on gut microflora, a novel colistin resistance mechanism was identified, which involved the expression of plasmid-encoded phosphoethanolamine transferase by the mobilized colistin resistance gene (*mcr1*). The resulting transfer of a phosphoethanolamine to lipid A on the cell membrane subsequently led to reduced colistin affinity, which led to resistance. The *mcr1* gene is mainly detected in *Escherichia coli* or *Klebsiella pneumoniae* isolated from a urine or blood sample of patients admitted to the hospital. The *mcr1* gene in the gut microflora was identified in over 19 countries, and the reported level per country was within the range of 1.4–2% [29]. For *A*. *baumannii*, however, the association between microbial resistance and the *mcr1* gene has not been reported. In this study, the resistance gene search using the CARD database showed that no mutation was detected for the following genes: *lpxA*, *lpxC*, *lpxD*, *pmrA*, *pmrB*, and *mcr1*.

In a comparative genomic analysis using NGS, the whole-genome sequences are compared with the reference genome, whereby a causal gene for a distinguished phenotype is detected, or the genomic characteristic shared by a specific group is identified. In this study, a comparative genomic analysis of the colistin-resistant strain was performed using the colistin-susceptible strain as the reference genome. The result showed that 57 CDS exhibited differences, among which 13 had a known name and function. These CDS contained 21 genes (*cysH*, *udg*, *ureC*, *DNMT1*, *dcm*, *tmk*, *DTYMK*, *glxK*, *garK*, *CHO1*, *pssA*, *mdh*, *udg*, *mutY*, *TST*, *MPST*, *sseA*, *cobS*, *cobV*, *ENO*, and *eno*) that may have an association with colistin resistance.

Among these genes, *tmk* and *DTYMK* are the genes engaged in dTMP kinase production, while being related to pyrimidine metabolism; *glxK* and *garK* govern the production of glycerate kinase and are related to glycerate activity; *CHO1* and *pssA* govern the production of CDP-diacylglycerol-serine O-phosphatidyltransferase and participate in cell membrane and lipid biosynthesis; *mdh* governs the production of malate dehydrogenase and participates in the oxidation of malate to oxaloacetate; *Udg* governs the production of uracil-DNA glycosylase (UNG), an enzyme that hydrolyzes the N-glycosylic bond between the uracil base and the deoxyribose sugar in the DNA containing uracil, to create an apyrimidinic site; *mutY* governs the synthesis of adenine glycosylase and plays a role in correcting G-A mispairs and the error-prone DNA synthesis past GO due to an oxidative damage on guanine; *TST*, *MTST*, and *sseA* are the genes for thiosulfate sulfurtransferase that transfer a sulfur moiety from thiosulfate to thiophilic acceptor; *cobS* and *cobV* are the genes engaged in adenosylcobinamide-GDP ribazoletransferase production and are related to the biosynthesis of cell membrane, inner membrane, cobalamin, and magnesium; *ENO* and *eno* are the genes engaged in the production of phosphopyruvate hydratase that catalyzes the reversible conversion of 2-phosphoglycerate to phosphoenolpyruvate, which is essential in carbohydrate decomposition during glycolysis. While these genes have not been reported to show an association with colistin resistance, there is a potential association. Notably, the genes for the biosynthesis of the cell membrane or lipids, such as *CHO1* and *pssA*, or the genes for the synthesis of the cell membrane and magnesium, such as *cobS* and *cobV*, are likely to be associated with colistin resistance as the target of colistin is the bacterial cell membrane and the mechanism of colistin action is to destabilize LPS by removing magnesium to cause cell death. Further studies should thus be conducted regarding these genes and their association with colistin resistance. In addition, there remains a possibility that, among the CDS that differ based on comparative genomic analysis, a novel gene without a known name or function is associated with colistin resistance. This is meaningful in suggesting the possibility that CDS, which shows a difference between the 27 colistin-resistant *A*. *baumannii* group and the colistin-susceptible standard strain, may be involved in the resistance of colistin. Further study is needed to define the association these CDS and colistin resistance.

In this study, molecular epidemiology was analyzed using whole-genome sequencing. The result of wgMLST showed two major clusters for the colistin-resistant *A*. *baumannii* strain. However, the two clusters exhibited no difference in the time of detection for each cluster, the samples, the pattern of antibiotic resistance, or the clinical epidemiology. In addition, a phylogenetic analysis was performed using SNP, and as in wgMLST, two major clusters were found, while several strains showed a deviation in classification. In the conventional MLST, the typing result showed ST1809, ST451, ST191, ST1837, ST369, and ST110, without any relation with the results of wgMLST and SNP analyses. The multidrug-resistant or carbapenem-resistant *A*. *baumannii* strains belong to the global clone 1 (GC1, formerly known as European clone 1 or international clone 1) and the global clone 2 (GC2, formerly European clone 2 or international clone 2) that are distributed worldwide. GC2 is more commonly distributed in Korea, and *A*. *baumannii* ST208, ST357, ST75, ST191, ST137, ST138, ST784, ST451, ST229, ST369, ST357, and ST552, have been reported [30–35]. This study examined ST191, ST451, and ST369, all of which belong to GC2.

The method of typing using the whole genome is known to ensure excellent results in analyzing an outbreak compared to the conventional methods. Of note is the study by Fitzpatrick et al., where a case of hospital outbreak infection with an *Acinetobacter* species was investigated. For the 148 strains (including 116 *A*. *baumannii* strains) isolated from the blood of patients, the following methods were compared: pulsed-field gel electrophoresis (PFGE), repetitive extragenic palindromic-PCR, the conventional method of typing, and the method using whole-genome sequencing (SNP analysis). Using the conventional MLST as the standard method, the conformity was 39% for PFGE, 65% for Rep-PCR, and 100% for whole-genome sequencing [36].

Regarding the molecular epidemiology of *A*. *baumannii*, further studies should compare the accuracy of wgMLST and SNP analysis. In this study, the results of wgMLST and SNP typing showed differences, but these methods may be useful in the strain typing analysis of *A*. *baumannii*. The differences between wgMLST and SNP analysis may be due to these factors; wgMLST analyses consider recombination as a single mutational event, whereas the SNP calling analysis considers each SNP in the recombinant segment as an additional mutation. However, as the wgMLST scheme includes accessory genes, the presence/absence of these genes might impact the resulting phylogeny [37]. This study analyzed the molecular epidemiology of colistin-resistant *A*. *baumannii* for the first time in Korea.

Through whole-genome sequencing for resistance gene analysis and comparative genomic analysis, colistin resistance was potentially related to certain genes or CDS, but no mutation was detected concerning colistin resistance. This study defined the characteristics of colistin-resistant *A*. *baumannii* isolated from Korea. In addition, although it is not known to be related to colistin resistance to date, candidate genes that may be associated with colistin resistance were presented, thereby serving as a cornerstone of research to elucidate the mechanism of colistin resistance. To conclude, the molecular epidemiology of colistin-resistant *A*. *baumannii* was analyzed using whole-genome sequencing, and the findings may prove useful in devising plans to prevent the spread of resistant bacteria and control the infection.

## Supporting information

S1 Table Antimicrobial resistance genes identified using whole-genome sequencing in 27 colistin-resistant *A*. *baumannii*. (DOCX)

S1 Raw images (PDF)
