# Supplementary material for: Whole-genome sequencing for the characterization of resistance mechanisms and epidemiology of colistin-resistant Acinetobacter baumannii
Source: PLoS One. 2022 Mar 4;17(3):e0264335. doi: 10.1371/journal.pone.0264335 (PMC8896714; doi:10.1371/journal.pone.0264335)
Supplement: S1 Table — (DOCX) [file pone.0264335.s001.docx]

**S1 Table. Antimicrobial resistance genes identified using whole-genome sequencing in 27 colistin-resistant *A. baumannii***

| **AMR Gene Family** | **Gene name** | **Mechanism** | **Drug class** | **Number of isolates** |
| --- | --- | --- | --- | --- |
| AAC(3) | *AAC(3)-IIb* | inactivation | aminoglycoside | 0 |
| AAC(6') | *AAC(6')-IIc* | inactivation | aminoglycoside | 11 |
| AAC(6') | *AAC(6')-Ij* | inactivation | aminoglycoside | 0 |
| ANT(3'') | *aadA13* | inactivation | aminoglycoside | 9 |
| ANT(3'') | *aadA2* | inactivation | aminoglycoside | 0 |
| ADC  beta-lactamase | *ADC-18* | inactivation | cephalosporin | 27 |
| ANT(3'') | *ANT(3'')-IIb* | inactivation | aminoglycoside | 27 |
| APH(3') | *APH(3')-Ia* | inactivation | aminoglycoside | 15 |
| APH(3'') | *APH(3'')-Ib* | inactivation | aminoglycoside | 16 |
| APH(3') | *APH(3')-VIa* | inactivation | aminoglycoside | 0 |
| APH(6) | *APH(6)-Id* | inactivation | aminoglycoside | 16 |
| chloramphenicol acetyltransferase (CAT) | *catB8* | inactivation | phenicol | 10 |
| macrolide phosphotransferase (MPH) | *mphD* | inactivation | macrolide | 18 |
| OXA beta-lactamase | *OXA-133* | inactivation | cephalosporin; penam | 3 |
| OXA beta-  lactamase | *OXA-23* | inactivation | cephalosporin; penam | 23 |
| OXA beta-  lactamase | *OXA-420* | inactivation | cephalosporin; penam | 0 |
| OXA beta-  lactamase | *OXA-66* | inactivation | cephalosporin; penam | 27 |
| TEM beta-lactamase | *TEM-1* | inactivation | monobactam; cephalosporin; penam; penem | 15 |
| VIM beta-  lactamase | *VIM-2* | inactivation | carbapenem; cephalosporin; cephamycin;  penam; penem | 0 |
| multidrug and toxic compound extrusion (MATE) transporter | *abeM* | efflux | fluoroquinolone; acridine dye; triclosan | 27 |
| small multidrug resistance (SMR)  antibiotic efflux pump | *abeS* | efflux | macrolide;  aminocoumarin | 27 |
| resistance-nodulation-cell division (RND) antibiotic efflux pump | *adeA* | efflux | glycylcycline; tetracycline | 27 |
| resistance-nodulation-cell division (RND) antibiotic efflux pump | *adeB* | efflux | glycylcycline; tetracycline | 27 |
| resistance-nodulation-cell division (RND) antibiotic efflux pump | *adeC* | efflux | glycylcycline; tetracycline | 27 |
| resistance-nodulation-cell division (RND) antibiotic efflux pump | *adeF* | efflux | fluoroquinolone; tetracycline | 27 |
| resistance-nodulation-cell division (RND) antibiotic efflux pump | *adeG* | efflux | fluoroquinolone; tetracycline | 27 |
| resistance-nodulation-cell division (RND) antibiotic efflux pump | *adeH* | efflux | fluoroquinolone; tetracycline | 27 |
| resistance-nodulation-cell division (RND) antibiotic efflux pump | *adeI* | efflux | macrolide;  fluoroquinolone; lincosamide;  carbapenem; cephalosporin; tetracycline;  rifamycin; diaminopyrimidine; phenicol ; penem | 27 |
| resistance-nodulation-cell division (RND) antibiotic efflux pump | *adeJ* | efflux | macrolide;  fluoroquinolone; lincosamide;  carbapenem; cephalosporin; tetracycline;  rifamycin; diaminopyrimidine; phenicol; penem | 27 |
| resistance-nodulation-cell division (RND) antibiotic efflux pump | *adeK* | efflux | macrolide;  fluoroquinolone; lincosamide;  carbapenem; cephalosporin; tetracycline;  rifamycin; diaminopyrimidine;phenicol; penem | 27 |
| resistance-nodulation-cell division (RND) antibiotic efflux pump | *adeL* | efflux | fluoroquinolone; tetracycline | 27 |
| resistance-nodulation-cell division (RND) antibiotic efflux pump | *adeN* | efflux | macrolide;  fluoroquinolone; lincosamid;  carbapenem; cephalosporin; tetracycline;  rifamycin; diaminopyrimidine;phenicol; penem | 27 |
| resistance-nodulation-cell division (RND) antibiotic efflux pump | *adeR* | efflux | glycylcycline; tetracycline | 27 |
| resistance-nodulation-cell division (RND) antibiotic efflux pump | *adeS* | efflux | glycylcycline; tetracycline | 27 |
| major facilitator superfamily (MFS) antibiotic efflux pump | *Escherichia coli mdfA* | efflux | tetracycline;  benzalkonium chloride;  rhodamine | 3 |
| ATP-binding cassette (ABC) antibiotic efflux pump | *msrE* | efflux | macrolide;  streptogramin | 24 |
| major facilitator superfamily (MFS)  antibiotic efflux pump | *tet(B)* | efflux | tetracycline | 16 |
| 16S rRNA methyltransferase (G1405) | *armA* | target alteration | aminoglycoside | 24 |
| sulfonamide resistant sul | *sul1* | target replacement | sulfonamide;  sulfone | 9 |
| sulfonamide resistant sul | *sul2* | target replacement | sulfonamide;  sulfone | 14 |
| major facilitator superfamily (MFS) antibiotic efflux pump | *tetR* | target alteration;  antibiotic efflux | glycylcycline;  tetracycline | 16 |
