# Supplementary material for: Whole-genome sequencing for the characterization of resistance mechanisms and epidemiology of colistin-resistant Acinetobacter baumannii
Source: PLoS One. 2022 Mar 4;17(3):e0264335. doi: 10.1371/journal.pone.0264335 (PMC8896714; doi:10.1371/journal.pone.0264335)
Supplement: S1 Raw images — (PDF) [file pone.0264335.s002.pdf]

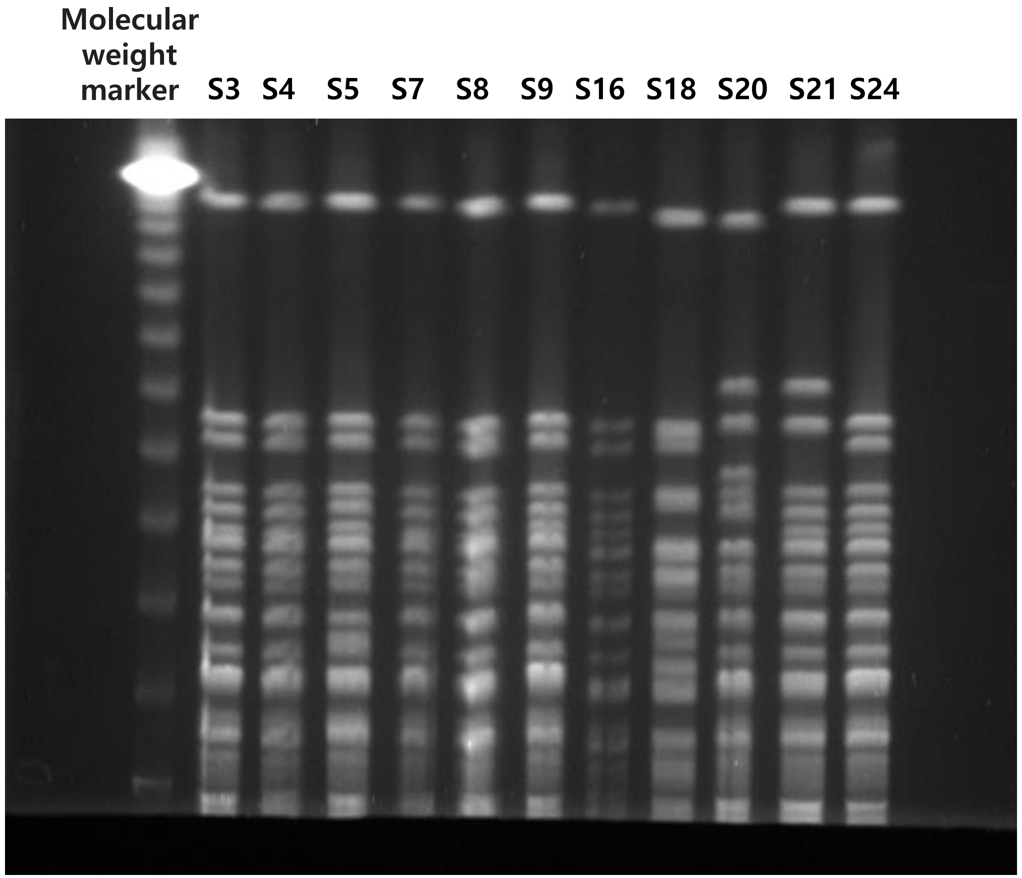
Molecular weight marker
S3
S4
S5
S7
S8
S9
S16
S18
S20
S21
S24

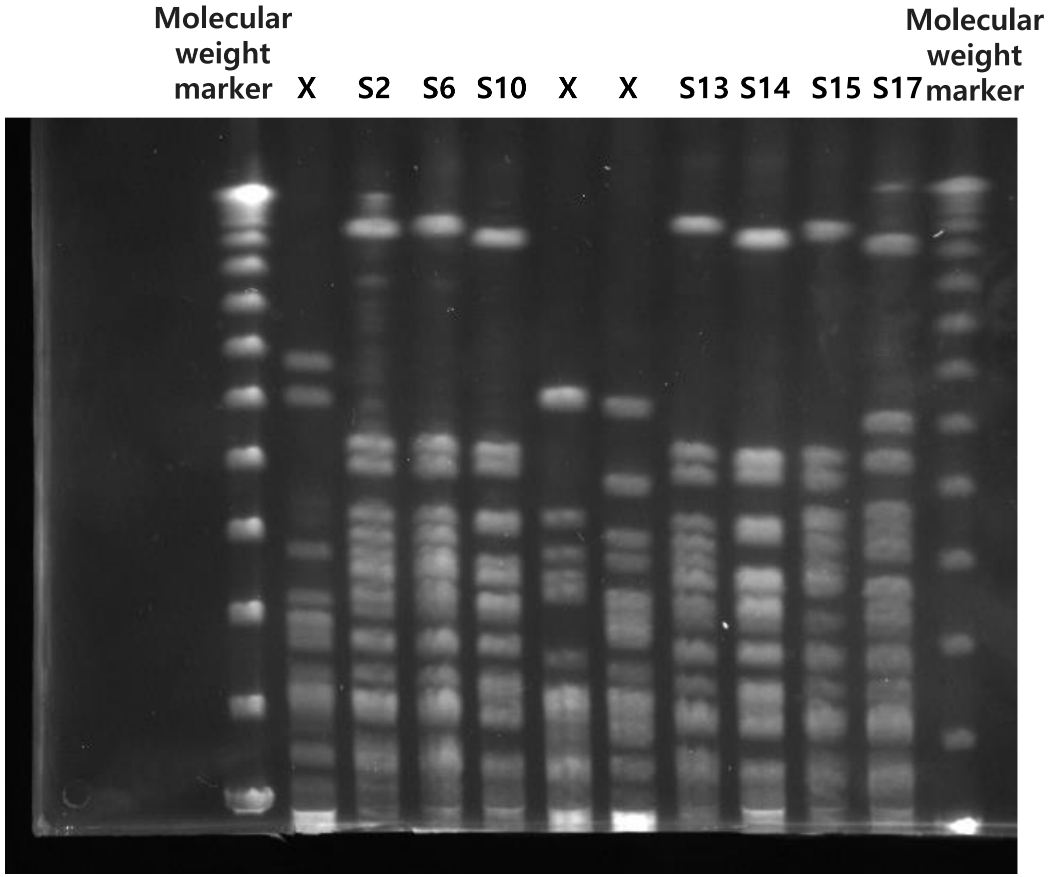
Molecular weight marker
X
S2
S6
S10
X
X
S13
S14
S15
S17
Molecular weight marker

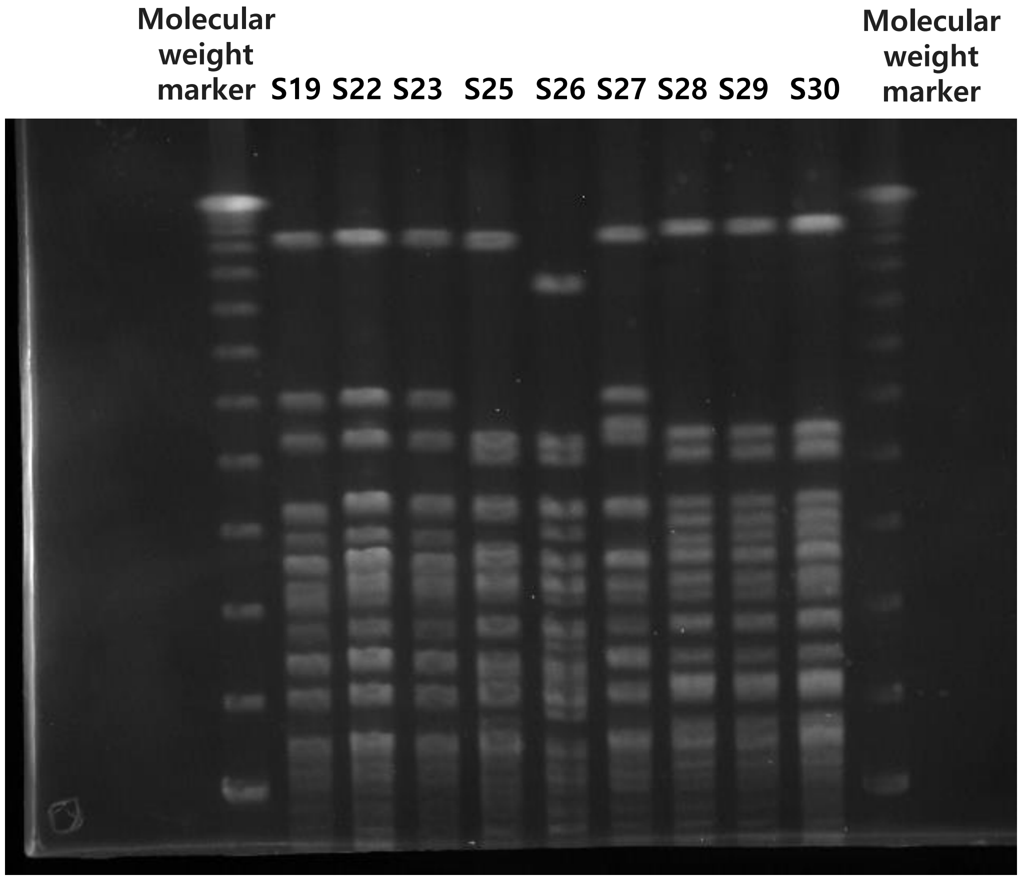
Molecular weight marker
S19
S22
S23
S25
S26
S27
S28
S29
S30
Molecular weight marker
